# Performance of Plain Woven Jute Fabric-Reinforced Polyester Matrix Composite in Multilayered Ballistic System

**DOI:** 10.3390/polym10030230

**Published:** 2018-02-26

**Authors:** Sergio Neves Monteiro, Artur Camposo Pereira, Carlos Luiz Ferreira, Édio Pereira Júnior, Ricardo Pondé Weber, Foluke Salgado de Assis

**Affiliations:** Materials Science Program, Military Institute of Engineering (IME), Praça General Tibúrcio 80, Urca, 22290-270 Rio de Janeiro, Brazil; snevesmonteiro@gmail.com (S.N.M.); camposo.artur@gmail.com (A.C.P.); cferreira@ime.eb.br (C.L.F.); edio@ime.eb.br (É.P.J.); rpweber@ime.eb.br (R.P.W.)

**Keywords:** jute fabric, polyester composite, multilayered armor, ballistic performance, economical advantage

## Abstract

The ballistic performance of plain woven jute fabric-reinforced polyester matrix composites was investigated as the second layer in a multilayered armor system (MAS). Volume fractions of jute fabric, up to 30 vol %, were mixed with orthophthalic polyester to fabricate laminate composites. Ballistic tests were conducted using high velocity 7.62 mm ammunition. The depth of penetration caused by the bullet in a block of clay witness, simulating a human body, was used to evaluate the MAS ballistic performance according to the international standard. The fractured materials after tests were analyzed by scanning electron microscopy (SEM). The results indicated that jute fabric composites present a performance similar to that of the much stronger Kevlar™, which is an aramid fabric laminate, as MAS second layer with the same thickness. The mechanism of this similar ballistic behavior as well as the comparative advantages of the jute fabric composites over the Kevlar™ are discussed.

## 1. Introduction

Ballistic protection of personnel, equipment and vehicles is nowadays of upmost importance due to armed urban conflicts and regional wars involving ever increasing fire power. In particular, the use of high velocity, impact and power (VIP) ammunition such as the class III [1] 7.62 × 51 mm (7.62 mm for short) constitute a major threat for personnel: either police officers, soldiers or civilians. In this case, single layered armor vests, such as those made only of steel or Kevlar™, Dupont, Wilmington, DE, USA, would require a large thickness, which interferes with the wearer mobility. For protection against VIP ammunition, the multilayered armor system (MAS) with a hard and brittle front material, like a ceramic, is an effective solution [2,3,4,5]. Traditionally, aramid fabric laminates such as Kevlar™ and Twaron™, Teijin, Arnhem, The Netherlands [2,3,4,5,6], as well as ultra-high molecular weight polyethylene (UHMWPE), such as Dyneema™ and Spectra™, Honeywell, Colonial Heights, VA, USA [7,8], are commercial materials used as MAS second layer. Another MAS third layer, normally a ductile metallic sheet, may be added to reduce even further the energy carried by the bullet impact shock wave [9]. In a ballistic test, the MAS is set as a target with a block of so-called clay witness standing behind. This block simulates a human body being protected by the MAS, and should only allow penetration of the fragments carried by the impact shock wave up to a standard limit of 1.73 in (44 mm) [1]. Beyond this depth of indentation in the clay witness, the ballistic test indicates a lethal trauma.

**Figure 1:**
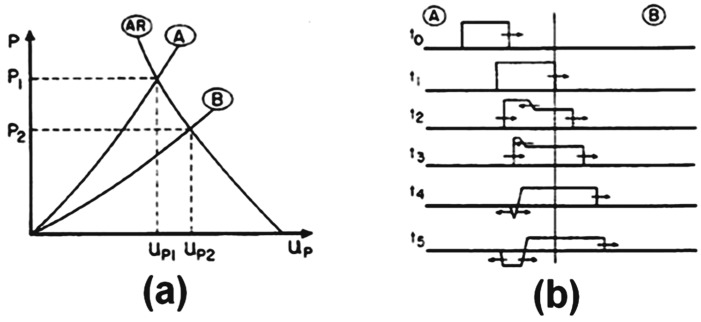
Transmission of shock wave from a medium with high to another with low density: (**a**) pressure-particle velocity plots; (**b**) stress profiles.

The efficiency of the MAS second layer involves a complex interaction with the shock wave and fragments coming from the initial bullet front impact. Indeed, the impact-generated compressive shock wave propagates inside the MAS and is partially reflected at each interface [3,10]. The density of the MAS materials affects the efficiency of energy absorption. Figure 1, adapted from Meyers [10], shows a schematic diagram of the pressure (*P*) variation with particle velocity (*U_p_*), after the impact of a projectile against a MAS front ceramic. The curves in Figure 1a reveals how the initial compressive shock wave pressure at *t*_0_ is affected by a second layer generating a reflected wave at *t*_4_, which travels back to the front layer. In these curves, A corresponds to the shock wave propagating in the front ceramic with higher impedance than B, the shock wave propagating in the second layer (Kevlar™ or jute fabric composite). At the A/B interface, a reflected tensile wave AR is associated with a relatively greater tensile pulse of pressure *P*_1_. In this case, the second layer has a lower density and the reflected tensile pulse at *t*_5_ in Figure 1b divides its energy with the smaller compressive wave moving ahead. Inside the first layer, the back-reflected tensile wave will efficiently contribute to shatter the brittle front ceramic, which can be more easily broken by a tensile pressure [11]. This process may be quantitatively evaluated by considering the materials shock wave impedance, *Z*, defined as

*Z* = ρ*U_s_* (1)

where ρ is the materials density and *U_s_* is the shock wave velocity in the material.

Based on the above-mentioned dynamic behavior of a MAS subjected to a ballistic impact, lighter material has been selected as the second layer. In particular, Kevlar™ and UHMWPE are nowadays common options. Analytical and material models were proposed to predict the ballistic efficiency of armors with Kevlar™ laminates [12] or UHMWPE filament-based composites [13]. However, less expensive natural materials could be used as MAS second layer. For instance, composites reinforced with natural lignocellulosic fibers are being extensively investigated as economical and sustainable alternatives [14,15,16,17,18,19,20,21] to replace synthetic fiber composites. Successful applications of natural fiber composites are taking place mainly in the automotive industry [22,23,24]. Another applied sector where natural composites are recently being investigated is ballistic armors [25,26,27,28,29,30,31,32,33,34]. Both natural fibers and fabrics reinforced polymer composites were found to present comparable ballistic performance to Kevlar™, when used in MAS second layer [28,29,30,31,32,33,34]. In particular, an epoxy composite reinforced with 30 vol % of jute fabric, employed as second layer of a MAS with front ceramic, successfully stopped the perforation of a 7.62 mm bullet [32]. The depth of indentation produced in a clay witness attended the standard [1] and was equal to that associated with MAS with Kevlar™ as second layer. In this previous work [32], however, the integrity of the jute fabric composite was not investigated nor was the shock wave impedance of the system.

**Figure 2:**
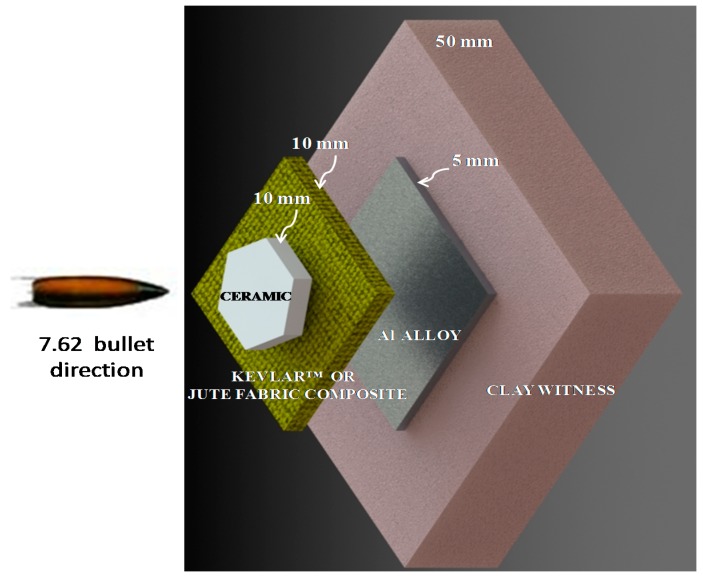
Schematic representation of the investigated multilayered armor placed ahead of a clay witness block.

In the present work, the ballistic performance of similar MAS, schematically illustrated in Figure 2, using as second layer either Kevlar™ or polyester composites reinforced with 10, 20 and 30 vol % of plain woven jute fabric was studied. Both the composite integrity and shock wave impedances are now evaluated. 

## 2. Experimental Procedure

**Figure 3:**
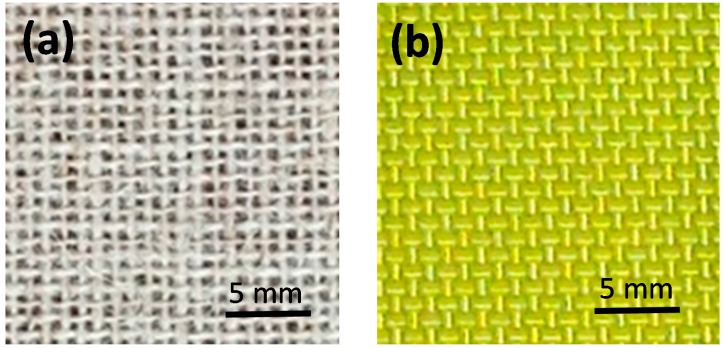
Plain woven fabrics of (**a**) jute and (**b**) aramid in Kevlar™.

The composites investigated as MAS second layers, Figure 2, were composed of jute or aramid plain woven fabrics, illustrated in Figure 3. The jute fabric, Figure 3a, with areal density of 345 g/m^2^, was supplied by Lealtex, Rio de Janeiro, Brazil. The aramid fabric, composing a model S745 Kevlar™, Figure 3b, with areal density of 460 g/m^2^, was acquired from LFJ Blindagens, São Paulo, Brazil. Kevlar™ laminates consists of 16 plies of aramid fabric bonded with 5 wt % of polychloroprene. 

Each MAS was composed of a front 10 mm thick Al_2_O_3_ 4 wt % Nb_2_O_5_ hexagonal ceramic tile with 31 mm of side dimension. The very brittle ceramic tiles were fabricated by mixing commercially pure Al_2_O_3_ powder, supplied by Treibacher Schleifmittel, Althofen, Austria, and Nb_2_O_5_ powder, supplied by the Brazilian Mining and Metallurgy Company, CBMM, Araxá, Brazil. The mixture was compacted at 30 MPa of pressure in a Nowak press, Nowak Comércio de Máquinas, São José do Rio Preto, Brazil. Sintering of the ceramic tiles was carried out in a model FE-1700 INTI electrical furnace, Maitec-Fornos INTI, São Carlos, Brazil, at 1400 °C for 3 h under air. The final ceramic grain size was about 4 μm.

Bonded to the front ceramic, a 150 × 120 × 10 mm composite plate second layer was produced by intercalating pieces of jute fabric with still fluid unsaturated orthophthalic polyester mixed with 1.0 wt % ethyl methyl ketone, as hardener, supplied by Resinpoxy, Rio de Janeiro, Brazil. After laying down in a steel mold the necessary amount of jute fabric and polyester for a final 10 mm thick plate, a pressure of 3 MPa was applied, by means of a Skay hydraulic press, Skay, São José do Rio Preto, Brazil, for 24 h. Each distinct composite required different numbers of jute fabric pieces. For 10, 20 and 30 vol % were used, respectively, 3, 6 and 9 pieces. A third layer of 150 × 120 × 5 mm 5052 H34 aluminum alloy sheet was also bonded to complete the MAS. Bonding was done with commercial Sikaflex™ polyurethane glue from Sika Brasil, São Pulo, Brazil.

Ballistic tests were carried out at the Army Center of Evaluation, CAEx, shooting range facility in the Marambaia peninsula, Rio de Janeiro, Brazil. The clay witness block, Figure 2, simulating a human body protected by the MAS, was placed in direct contact with the back aluminum alloy sheet. The special clay for this purpose is known as plastiline and was supplied by Corfix, Brazil. All ballistic tests were conducted according to the NIJ standard [1] using class III 7.62 × 51 mm army ammunition shot from a gun barrel located 15 m from the MAS target inside a CAEx tunnel. The 7.62 mm bullet (lead with 9.7 g) velocity was measured by optical barriers and Doppler radar. For each kind of tested material, 10 experimental runs were performed and mean values with corresponding standard deviations were obtained.

**Figure 4:**
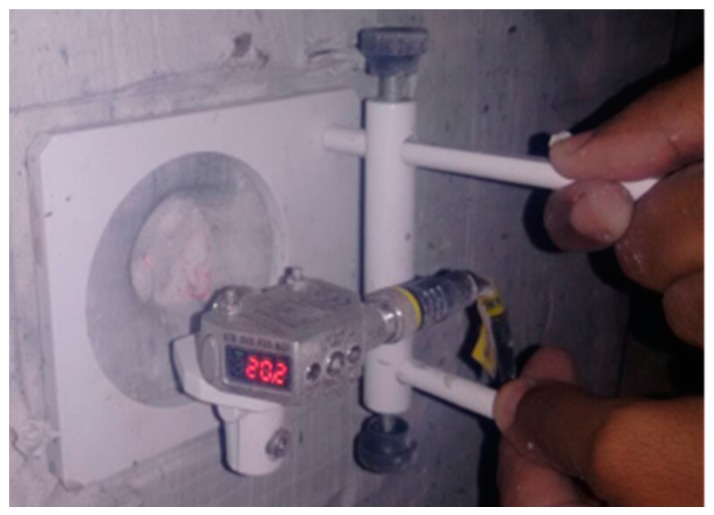
Depth of indentation in the clay witness measured with laser sensor caliper.

All ballistic tests did not completely perforate both the MAS and clay witness block. As a consequence, an indentation was produced in the clay. The depth of indentation duplicates the plastic deformation imposed on the aluminum sheet by the bullet impact. According to the standard [1], the measured depth of indentation is limited to 44 mm in order to avoid a lethal trauma to the MAS wearer. Measurements were performed with a laser sensor caliper with 0.01 mm of precision, as shown in Figure 4. The Weibull statistic was used to analyze the depth of indentation results.

Fractured pieces of MAS components dispersed, after ballistic tests, were analyzed by scanning electron microscopy (SEM) in a model Quanta FEG 250, FEI microscope operating with secondary electrons at 20 kV.

Shock waves impedances defined by Equation (1) were calculated using the matching analysis for all different types of MAS with jute fabric composites as second layer.

## 3. Results and Discussion

**Figure 5:**
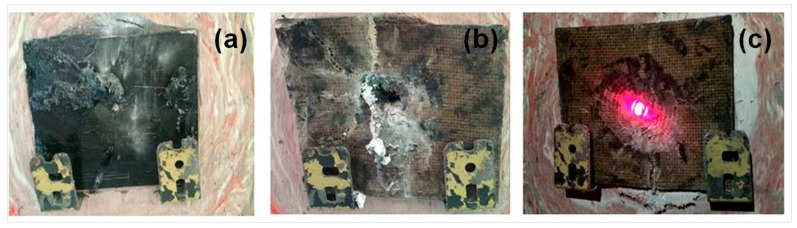
Typical aspect after ballistic test of multilayered armor system (MAS) targets with second layer of (**a**) 10 vol %, (**b**) 20 vol % and (**c**) 30 vol % jute fabric-reinforced polyester composite.

In all ballistic tests using jute fabric composites as MAS second layer, the impact energy failed to perforate the aluminum alloy third layer. This last layer was plastically deformed and caused a depth of indentation in the clay witness smaller than 44 mm, which is the required limit by the NIJ standard [1]. Figure 5 illustrates the aspect of the different MAS targets after the ballistic tests. In this figure, one sees that the hexagonal front ceramic has disappeared by complete shattering in all targets. Moreover, in the MAS of Figure 5a, its second layer of 10 vol % of jute fabric composite has also disappeared by complete disintegration. As for the MAS in Figure 5b, its second layer of 20 vol % of jute fabric composite was only partially disintegrated. The only second layer, Figure 5c, which remained intact was that of 30 vol % of jute fabric composite. The bright spot at the middle of the target in Figure 5c is due to the red laser sight for bullet precision.

Table 1 presents the average values and corresponding Weibull parameters for the measured indentation depths on the clay witness for the different MAS including that for Kevlar™ as second layer, which was also previously reported [28,29,30,31,32,33,34]. The relatively high values of the Weibull modulus β in Table 2 indicate unimodal and precise characteristic values for the measured depth of indentation in Table 1.

**Figure 6:**
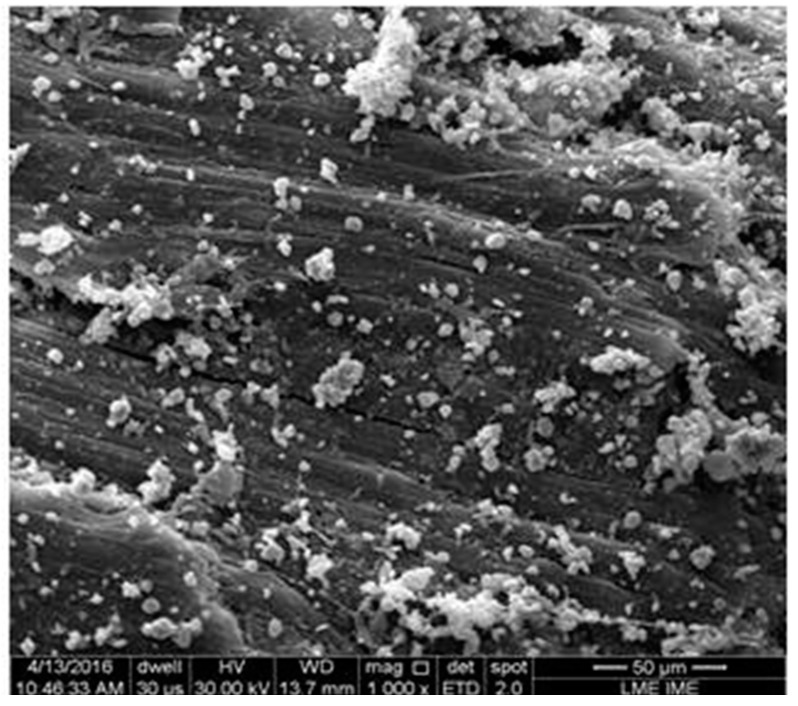
SEM of the fracture surface of 30 vol % jute fabric composite covered with ceramic fragments.

The results of depth of indentation in Table 1 are, within the standard deviation, practically equal. Although surprising, similar results were also found for other natural fiber/fabric [28,29,30,31,32,33,34] and Kevlar™ as second layer of MAS with the same dimensions, as the schematic displayed in Figure 2. The reason for this similar ballistic performance is the ability of the second layer, in a MAS with front ceramic, to collect fragments generated from the ballistic impact [35]. This ability does not require stronger fibers but mechanisms of mechanical incrustation as well as fragment attraction by Van der Waals forces and static charges on the fiber surface, of either synthetic Kevlar™ [35] or natural fabric [32,33]. Figure 6 shows the SEM fractograph illustrating the mechanism of fragments (white particles) capture by the 30 vol % jute fabric-reinforced polyester composite as MAS second layer.

The actual graphical representation of the impedance matching for the ballistic impact shock wave travelling from the front ceramic layer to the second jute fabric composite layer corresponds to that already shown schematically in Figure 1a. Owing to the comparatively lower density of the jute fabric composites, a tensile wave is reflected in Figure 1b, contributing to shatter the front ceramic, as shown in Figure 5. 

Table 2 presents the results of impedance matching analysis. In principle it would be expected that the lower the shock impedance of the second layer, the smaller the depth of indentation in the clay witness.

In this table, it is important to note that all jute fabric composites as MAS second layer have significantly lower values than Kevlar™. Among themselves, the jute fabric composites display relatively small differences in the value of Z. Therefore, one might infer that their ballistic protection should not be much different, as indicated in Table 1. Shock waves impedances defined by Equation (1) were calculated using the matching analysis for all different types of MAS with jute fabric composites as second layer. Actually, the lowest impedance value in Table 2 corresponds to that of the 10 vol % jute fabric composite. However, for practical purposes, it would not be the most convenient MAS second layer due to complete disintegration, as shown in Figure 5a, after the bullet impact. Therefore, in terms of personal protection, required by the standard [1], the impact energy of subsequent multiple shots will not be dissipated by this disintegrated second layer. Only the 30 vol % jute fabric composite, which remains intact after the bullet impact, Figure 5c, will guarantee protection against multiple shots.

In order to evaluate the impact energy dissipated by each jute fabric composite alone, i.e., separated from the MAS, special ballistic tests were carried out with a composite plate in front of a cylindrical metallic block with a hole. In these tests the velocities of the 7.62 mm bullet, before and after perforation of the composite plate were measured. The impact velocity, *V_i_*, and residual velocity, *V_r_*, allow the calculation of the energy ∆*E_d_* dissipated inside the composite

(2) $$\Delta E_{d}=\frac{1}{2}m\left(V_{i}^{2}-V_{r}^{2}\right)$$

where *m* = 9.7 g is the lead bullet mass.

**Figure 7:**
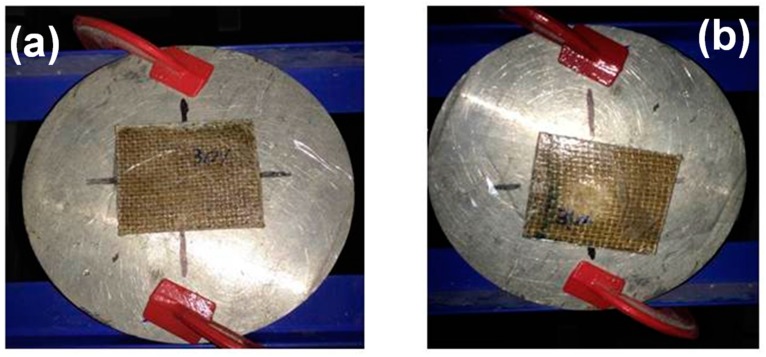
Ballistic test to measure the impact and residual velocities after perforation of a jute fabric composite plate: (**a**) before and (**b**) after bullet impact.

Figure 7 shows a typical test for residual velocity in a plate of 30 vol % jute fabric-reinforced polyester composite. In Figure 7a, the composite is placed in front of the hole of the metallic block before the ballistic test. After the test, a perforation is revealed in the composite, as shown in Figure 7b, due to the bullet impact.

Table 3 presents the impact and residual velocities as well as the internally dissipated energy, Equation (2), from ballistic tests of individual jute fabric composites. This table also presents results from the Al_2_O_3_ 4 wt % Nb_2_O_5_ ceramic and Kevlar™ obtained elsewhere [32]. One should notice in Table 3 that more than 70% of the energy dissipation occurred in the ceramic, which agrees with previously reported results [35]. By contrast, individually, the other MAS components dissipate less than 10% of the bullet energy in each one. In particular, the Kevlar™ dissipates a very low amount of energy (less than 3% in comparison to those dissipated by the jute fabric composites). To some extent, these results are coherent with the ones in Table 1, where Kevlar™ has practically the lowest ballistic performance as compared to those of the jute fabric-reinforced polyester composites in terms of depth of indentation. In spite of the relatively higher energy absorbed (Table 3) by the 10 vol % jute fabric composite as MAS second layer, only the 30 vol % jute fabric composite, which remained intact (Figure 5c) after the bullet impact, would attend the standard [1] for personal vest protection against multiple shots. 

The relatively low energy dissipation by the Kevlar™ alone in Table 3 is a consequence of the high VIP ammunition used in the test. A sharp-pointed 7.62 mm projectile penetrates easily between the aramid fibers in the Kevlar™. This causes breaking and stretching as well as separation and pullout of the fibers [6]. However, both the bullet and the front ceramic in the MAS are reduced to fragments after the bullet impact. In this case both Kevlar™ and natural jute fiber fabric composites, as MAS second layer, are efficient barriers to the remaining ballistic energy by capturing the fragments [28,29,30,31,32,33,34].

Although Kevlar™ and jute fabric composites in Table 1 display practically the same ballistic performance, the jute fabric, as any natural-based material, is also associated with environmental and societal benefits [36]. Nowadays, these advantages contribute to a practical indication that armor vests using 30 vol % of jute fabric-reinforced polyester composite, as MAS second layer, might be more convenient than Kevlar™ or even other jute fabric composites.

## 4. Conclusions

- Plain woven jute fabric-reinforced polyester composites, used as second layer of a multilayered armor system (MAS) with front ceramic and back aluminum alloy sheet, attended the international ballistic standard.
- The depth of indentation in a clay witness simulating a human body protected with a MAS as target against high velocity 7.62 mm bullet was practically the same, within the statistical precision, in the jute fabric composites and the Kevlar™ used as MAS second layers.
- Mechanisms of ceramic and bullet fragments capture are equally efficient for Kevlar™ and jute fabric composites. This is also verified in the values obtained for shock wave impedance of these materials.
- In spite of similar ballistic performance, the 30 vol % jute fabric-reinforced polyester composite, which is not destroyed after impact, has environmental and societal advantages that justify its substitution for Kevlar™ or other jute fabric composites as MAS second layer.

## Figures and Tables

**Table 1:** Depth of indentation for different investigated multilayered armor systems.

MAS Target with Second Layer	Depth of Indentation (mm)	Modulus (β)	Precision (R^2^)
10 vol % jute fabric polyester composite	18 ± 3	6.81	0.96
20 vol % jute fabric polyester composite	23 ± 3	9.29	0.94
30 vol % jute fabric polyester composite	17 ± 2	9.76	0.98
Kevlar™	21 ± 3	8.43	0.90

**Table 2:** Calculated parameters for the impedance matching analysis.

Second Layer at Ceramic Interface	U_p_ (m/s)	P (GPa)	U_s_ (m/s)	Z (10^6^ kg/m^2^s)
Polyester-10 vol % jute fabric	741	2.02	2440	2.74
Polyester-20 vol % jute fabric	733	2.34	2798	3.19
Polyester-30 vol % jute fabric	724	2.61	3105	3.60
Kevlar™	715	2.99	2909	4.19

**Table 3:** Impact and residual velocities together with internally dissipated energy in individually ballistic tested MAS components.

MAS Component	V_i_ (m/s)	V_r_ (m/s)	E (kJ)	ΔE_d_ (%)
Al_2_O_3_ ceramic	848 ± 6	567 ± 43	1.93 ± 0.310	71.75
10 vol % jute fabric polyester composite	838 ± 3	805 ± 7	0.26 ± 0.004	9.67
20 vol % jute fabric polyester composite	837 ± 4	807 ± 5	0.24 ± 0.006	8.92
30 vol % jute fabric polyester composite	837 ± 8	812 ± 8	0.20 ± 0.008	7.44
Kevlar™	848 ± 6	841 ± 7	0.06 ± 0.001	2.23
